# Changes in ventricular depolarisation vectors during exercise caused by regional myocardial ischaemia

**DOI:** 10.1038/s41598-019-52869-0

**Published:** 2019-11-08

**Authors:** Cameruddin W. Vellani, Satwat Hashmi, Sadia Mahmud, Mohammad Yusuf, Safia Awan, Khawar Kazmi

**Affiliations:** 1 Section of Cardiology, Department of Medicine, The Aga Khan University, Stadium Road, P.O. Box 3500, Karachi, 74800 Pakistan; 2 Department of Biological and Biomedical Sciences, The Aga Khan University, Stadium Road, P.O. Box 3500, Karachi, 74800 Pakistan; 3 Department of Medicine, The Aga Khan University, Stadium Road, P.O. Box 3500, Karachi, 74800 Pakistan; 4 Department of Radiology, The Aga Khan University, Stadium Road, P.O. Box 3500, Karachi, 74800 Pakistan

**Keywords:** Physiology, Cardiology

## Abstract

Research at the Aga Khan University for several years has been directed to find a reliable, low-cost, portable, non-invasive method for identification of coronary artery disease, its location and extent. A new method has been devised to measure the magnitude and direction of cardiac electrical vectors in three perpendicular planes during physical exercise to identify reduction in myocardial excitability as the electrophysiological marker of hypoxia. This report shows that changes in electrical forces due to exercise-induced regional hypoxia serve as indicators of reversible myocardial ischaemia. Changes in the magnitude and direction of vectors at stages of the Bruce protocol were measured in healthy volunteers, and patients undergoing the same exercise protocol for distribution of a radioactive tracer injected intravenously at peak exercise and after recovery (myocardial perfusion scan). Alterations in the magnitude and direction of resultant vectors during exercise were scored to enable analysis. Analysis identified slow progression of myocardial depolarisation as the electrophysiological marker of regional hypoxia relative to physical work. Compared with myocardial perfusion scan the sensitivity and specificity of electrical vectors for identification of ischaemia were 88% and 71%, respectively. Accuracy of ischaemia shown by electrical vectors is being assessed in patients undergoing elective coronary angiography.

## Introduction

Diverse clinical presentations of insufficient tissue perfusion for myocardial work, hereafter referred to as myocardial ischaemia, particularly the chronic, stable, asymptomatic state, are common and often difficult to assess reliably without complex non-invasive special investigation requiring expensive installations in urban institutions and high cost for patients. Consequently, access is difficult for middle and low-income populations in economically developing countries where cardiovascular disease is common^1^.

A portable, low-cost method of analysing electrical vectors during exercise has been developed at the Aga Khan University (AKU), based on electrophysiological effects of hypoxia on myocardial depolarisation. The method was patented (Vectorcardiographic Signal Analyzer; United States Patent Numbers: US 9, 226, 674 B2, January, 2016 and 9,560,981 B2, February 2017).

Trial of the method^2^ in patients referred for myocardial perfusion scan (MPS) compared with young, healthy volunteers undergoing a standard exercise tolerance test (ETT) showed significant differences in the magnitudes of vectors between patients and volunteers.

Changes in the magnitude and direction of electrical vectors during treadmill exercise were studied in larger groups of patients and healthy young volunteers in order to identify changes that indicate myocardial ischaemia by comparison with reversible defects in perfusion shown by MPS. The methods, results and inference of this study are reported in this paper.

## Methods

The study was carried out in accordance with relevant guidelines and regulations of the Aga Khan University and its Ethical Review Committee (ERC). All experimental protocols were approved by the Aga Khan University ERC and informed consent was obtained from all subjects.

ECG signals were recorded by orthogonal leads from male patients referred by cardiologists for assessment of myocardial ischaemia by MPS during treadmill exercise and consented to participate in the research study.

The number of sequentially recruited patients was 140. The data of 118 patients and 51 volunteers, total subjects 169, were available for statistical comparison of changes in the magnitude and direction of vectors. The exclusion of 22 patients from the study was due to signal artefacts in one or more leads causing inconsistent computation by the software for analysis of electrical vectors, or insufficient data available in any lead for comparison of changes in vectors.

The patients studied were aged 36–77 years (mean 55.3 ± 8.1); the means and standard deviation of their BMI were 28 and 4.42, respectively. The frequencies of co-morbidities were: diabetes mellitus 34%; hypertension 60%; hyperlipidaemia 25%; smoking 20%. The mean and standard deviation of their heart rates (HR) and blood pressures (BP) were: before exercise, HR 72 ± 14/min, BP 134 ± 19/78 ± 9 mm Hg; at peak exercise, HR 147 ± 20/min, BP 167 ± 24/83 ± 10 mm Hg. Patients exercised up to their level of tolerance or attainment of the target heart rate in accordance with the Bruce protocol for a standardized multistage treadmill exercise stress test for assessing patients with suspected coronary artery disease.

The standard ECG and orthogonal lead signals of healthy male volunteers aged 17–27 years (mean 22.6 ± 2.9), BMI mean 22.1 ± 3.6, during treadmill exercise in accordance with the Bruce protocol were also studied. Volunteers were recruited from staff of AKU or youth known to them in the community who exercised regularly, were asymptomatic, did not take any drugs or smoke or chew tobacco. The ranges of their heart rates and blood pressures were: before exercise, HR 73 ± 12/min, BP 117.64 ± 4.14/77.80 ± 4.66 mm Hg; at 9 minutes of exercise, HR 172 ± 8/min, BP 158 ± 14/80 ± 3 mm Hg. All volunteers exercised to stage 4 of the protocol. Male subjects only were studied because of difficulty in recruiting female patients and volunteers.

Considering the results of the first study^2^, we intended to confirm the association of reversible reduction in vector magnitude during exercise with reversible defects in regional perfusion shown by MPS, and determine whether enhancement of the magnitude and changes in direction of vectors, together with reduction in magnitude, could provide specific criteria for ischaemia induced by exercise.

The required sample size was calculated for a repeated measures study design^3^. In order to detect a difference of one standard deviation for reduced and enhanced relative vector magnitude (RVM), and change in angle scores between groups of patients with and without ischaemia shown by MPS, at least 21 subjects were required in each group. The parameters were 80% power and at 5% significance level, assuming that the minimum correlation among the scores for one subject is 0.5 and allowing for the interaction between exercise stage and the groups of patients.

MPS was the only reliable test for myocardial ischaemia induced by exercise used for all patients of the study. The radio-active tracer, Tc-99m Tetrofosmin, was given intravenously at peak exercise for stress images and after recovery for the rest images. Images of the distribution of the tracer were recorded by trained technologists. The scans were reported by cardiologists trained in nuclear imaging. The reporters were not privy to the analysis of electrical vectors.

The 12-lead ECG records of patients and volunteers, both obtained at rest and at each stage of the Bruce protocol for ETT, were examined by an experienced cardiologist and a physiologist for changes in the ST segment indicative of ischaemia, independent of the reports given by the clinical service. The ECG criterion for ischaemia was reversible depression of the QRS-ST junction and ST segment at 80 ms after the junction by 0.1 mV or greater.

Recording of electrical signals by two sets of amplifiers required placement of 10 adhesive electrodes of 3 orthogonal bipolar leads for derivation of electrical vectors (vectorcardiography (VCG)) and 10 for monitoring 12 leads of the standard ECG.

The signals of 3 orthogonal leads, based on the system of McFee and Parungao^4^, were recorded simultaneously, digitised and stored for later computation. Analysis of the data by software developed at AKU involved averaging 30–80 accurately aligned digitised data of ventricular depolarisation^2^. The averaged lead signals represented consistent elements of the QRS waveform at each stage of the Bruce protocol for treadmill exercise and provided the coordinates of vectors at intervals of 1.5 millisecond (ms) in frontal, horizontal and sagittal planes. Numerical values of the magnitude and direction of the planar electrical vectors at stages of increasing effort were provided in Excel for further computation.

### Reference for measurement of change in vectors

During analysis of the data of volunteers and patients we noted changes in the magnitude and direction of vectors as early as 0.5–1 minute (e1) of exercise from the values just before commencement of exercise. At 2.5–3 minutes of exercise (e3) the trends of vector magnitudes and directions were similar to e1 but varied often at 5.5–6 min (e6) and more so at 8.5–9 min (e9). Therefore changes in characteristics of vectors were measured with reference to the relative stability of measurements at e1.

### Representation of measurements by scores

The extent of change in the magnitude of a vector at a stage of exercise and e1 was represented as a proportional value because the magnitudes of vectors vary considerably across the period of depolarisation. A difference of 0.1 in proportion indicated a change of 10% in the magnitude; this was represented by a score of one unit, in order to accommodate extraneous effects induced by noise. In this paper, reduction and enhancement of the relative vector magnitude are expressed as RVM− and RVM+, respectively.

The extent of change in direction of a resultant vector was indicated by the averaged absolute differences of the 3 planar vector angles; a displacement of 10 degrees was represented by 1 unit. Representative scores of changes in magnitude and direction greater than 5 units were not considered because changes greater than 50% in RVM and 50 degrees in direction indicated gross change in the sequence of depolarisation without a pointer to a specific cause.

### Location of reduction in electromotive forces (EMF)

The orientation of vectors was determined relative to the virtual intersection of the 3 orthogonal lead axes that was estimated to occur in the cavity of the left ventricle approximately 25% of the distance from apex to the aortic valve.

The orientations of vectors at e1 whose magnitudes were subsequently altered during exercise indicated the spatial location of change in EMF. The clockwise or anticlockwise displacement of changed planar vectors implied reduction of EMF in the opposite direction; this inference provided an additional indication of the regional location of altered vectors.

### Statistical analysis

In both patients and healthy young volunteers reduction of RVM occurred for short periods during exercise, generally in the first half of depolarisation. These short periods implied regional changes that were expected in ischaemia, therefore it seemed appropriate to consider differences in summed scores of reduced or enhanced RVM and change in direction separately in four successive periods of 20 ms. However, when the changes in contiguous vectors crossed into the next 20 ms period the scores were summed over both periods.

The scores for changes in resultant magnitudes and averaged planar directions of vectors, summed in each of four consecutive 20 ms periods of depolarisation at each of the 3 exercise stages (e3, e6 and e9), were examined by Univariate analysis; planar vector magnitudes and extent of displacement, (clockwise (+) or anticlockwise (−) in accordance with a left-handed frame of reference) were examined by Multivariable analysis.

For Multivariable analysis, the first two 20 ms periods of depolarisation were collapsed as one category versus the last two periods. The predictor variables considered were group (patient versus volunteers), exercise stage (e3, e6 and e9) and depolarisation periods (second half versus the first half).

The histograms for each outcome in all 3 planes showed a zero-inflated data structure. The non-zero measurements formed a skewed distribution. A Probit log-skew-normal Mixture model was used for adequate analysis of zero-inflated data for repeated measurements. The Mixture model^3,5^ has two parts; the first part considers the outcome as binary, the second part involves continuous log-skew-normal distribution. For this study clinical interest lay in the second part of the model, regarding non-zero values of each outcome. Details of the analyses, histograms and results of the second part are reported in Supplementary Material-2.

## Results

### Differences in vectors between groups of subjects during exercise

Univariate analysis of RVM and direction of vectors showed greater changes in patients than healthy volunteers (Supplementary Material 1, Table S1) at all stages of exercise, especially in the initial 40 ms of depolarisation at 5.5–6 minutes; *p* values for reduced RVM and change in direction at 21–39 ms were 0.001 and 0.01, respectively.

The possible effect of age and fitness for exercise on changes in vectors was examined by comparing the group of patients who had MPS evidence of ischaemia (age 60.5 ± 7 years) with the group without past or current evidence of myocardial ischaemia (age 52.8 ± 8 years). The results are given in Table S2 of Supplementary Material-1. Despite the marked variance of data, at 5.5–6 minutes of exercise the mean score of reduced RVM in patients with positive MPS was marginally greater at 21–39 ms, *p* value 0.08, and more significantly at 40.5–58.5 ms, *p* value 0.03. Differences between group mean scores for enhanced RVM and change in direction at 21–39 ms were clear in the first 40 ms of depolarisation, *p* values 0.01 and 0.002, respectively. These significantly greater changes in vectors of the group with reversible perfusion defects shown by MPS (MPS +ve) indicates inducible changes in the sequence of depolarization with exercise.

Multivariable analysis was done in order to find reliable indicators of ischaemia. Scores representing reduced and enhanced RVM, and direction of displacement of planar vectors during exercise provided adjusted analysis of differences between patients and healthy young volunteers.

The results of the second part of the Mixture model are outlined below (The prefix S refers to Table numbers in Supplementary Material 2).

1. Reduced RVM: at any stage of exercise, reduction of RVM was significantly greater for patients than volunteers in the first half of depolarisation in the horizontal plane, and in either half of depolarization in frontal and sagittal planes (Tables S3, S4 and S5).
2. Enhanced RVM: in the 3 planes enhancement was significantly greater with exercise in patients than volunteers and in both occurred mainly in the second half of depolarisation (Tables S6–10).
3. Anticlockwise displacement of vectors: in the horizontal and frontal planes, displacement increased with exercise, greater in the second half of depolarisation (Table S11), and significantly greater in patients than volunteers (Tables S12 and S13). In the sagittal plane, anticlockwise displacement increased with exercise, greater in the first half of depolarisation than the second (Table S14), marginally greater in patients than volunteers.
4. Clockwise displacement of vectors: in the 3 planes, displacement increased significantly with exercise in patients and volunteers, without significant difference between them. In the horizontal plane, displacement was greater in the first half of depolarisation than the second (Table S15). In the frontal plane, displacement was significantly greater in the second half of depolarisation than the first. In the sagittal plane, displacement was highly significant (*Wald p* values < 0.0001) in both patients and volunteers, in either half of depolarization (Table S16).

### Deductions from the multivariable analysis

- Similar changes in magnitudes and displacement of electrical vectors occur with exercise in patients and healthy young volunteers but with differences between the first and second 40 ms of depolarisation; this implies differences in electrical properties (unspecified) of myocardial regions that are depolarised in the two periods.
- Greater reduction in the magnitude of vectors during exercise in patients than healthy volunteers was confirmed but not shown to be a specific indicator of myocardial ischaemia.
- Enhancement of magnitudes and displacement of vectors with exercise, greater in patients than volunteers, occur mainly in the second half of depolarisation, implying alteration in the sequence of depolarisation.
- Anticlockwise displacement of vectors in the horizontal and frontal planes was significantly greater in patients than volunteers in the second half of depolarisation, implying greater reduction in progression of depolarisation that could be an indicator of ischaemia. Clockwise displacement showed no difference between the two groups.

### Identification of myocardial ischaemia in individuals

Variations in the trends of resultant vector magnitudes at stages of exercise relative to e1 showed that delay or failure in progression of a trend leads to subsequent difference of magnitudes relative to the corresponding vectors at e1. Consequently, RVM is not a reliable measure of change but could serve as a marker of change.

The rate of progression of the resultant vector magnitude at stages of exercise is given by differences between the voltages of consecutive vectors sampled at a constant interval. This parameter is equivalent to the first derivative of the trend that measures the rate of progression of depolarisation. Differences between consecutive vectors, expressed as proportions of the corresponding values at e1, indicate the severity of reduction and its duration, as well as variation with the stages of exercise.

**Figure 1:**
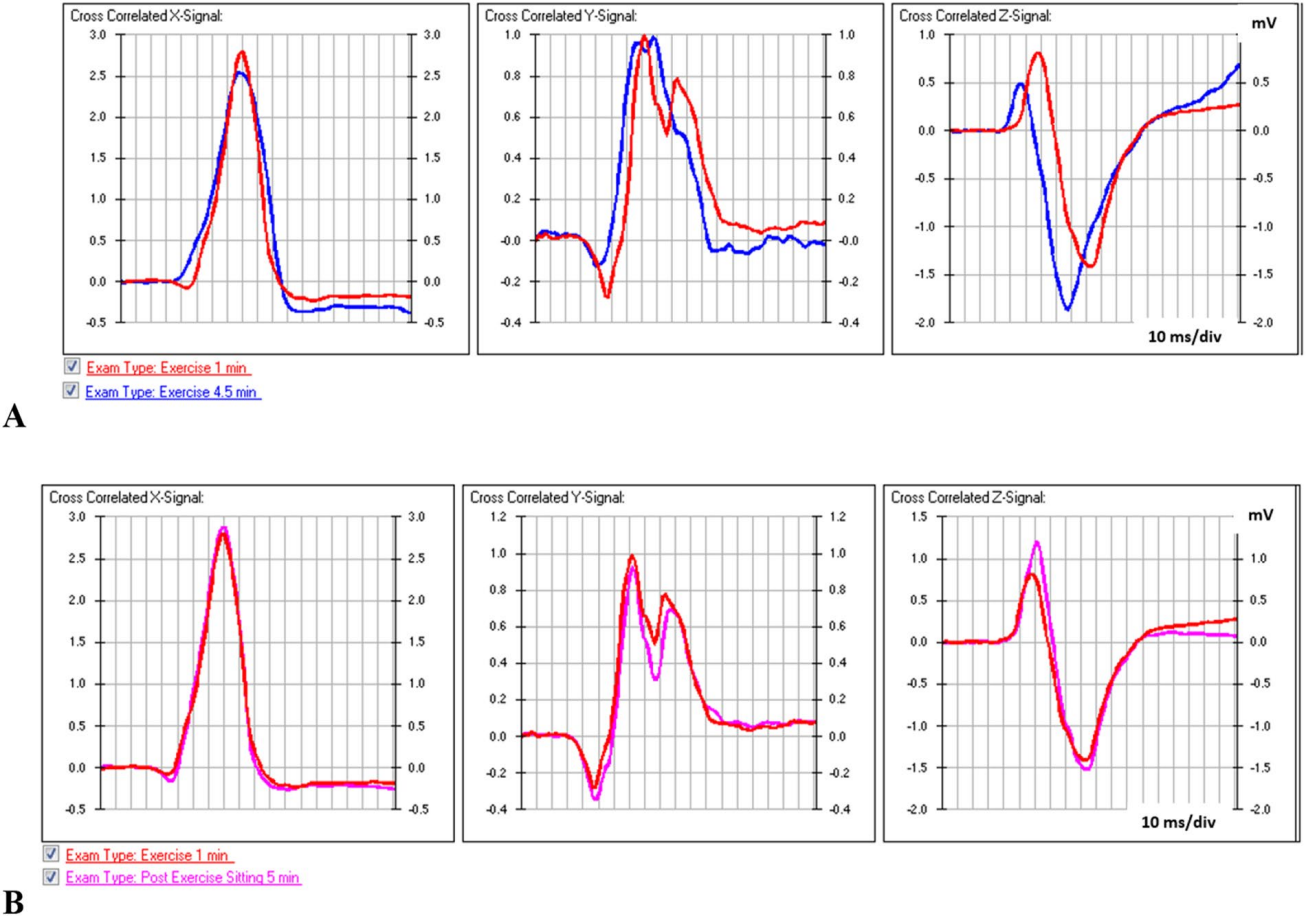
Aligned orthogonal lead waveforms of ventricular depolarisation. (**A**) Recordings at one minute and 4.5–5 minutes of exercise and (**B**) at rest 5 minutes post-exercise.

**Figure 2:**
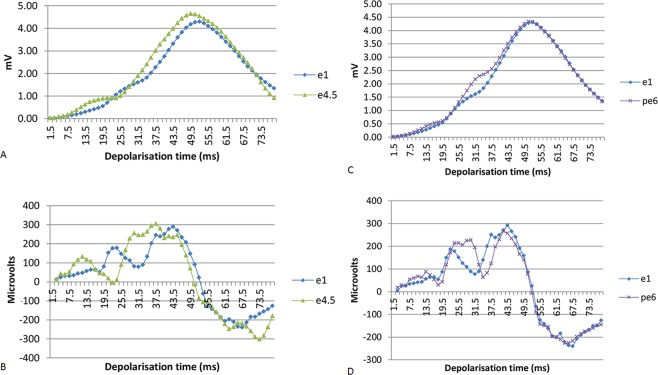
Magnitudes of resultant vectors of a patient (P201) during and after exercise. Identification of myocardial ischaemia by changes relative to the first minute of exercise (e1), 4.5–5 minutes of exercise (e4.5) and 6 minutes post exercise (pe6). (**A**) Vector magnitudes, exercise (e) 1 min and 4.5 min. (**B**) Differences between vector magnitudes on exercise. (**C**) Vector magnitudes post-exercise (pe). (**D**) Differences between vector magnitudes post-exercise.

The relationship and reversible nature of RVM and rate of progression of vector magnitude due to ischaemia, as well as its location are evident in the data of a patient (P201) illustrated in Figs 1 and 2.

This patient exercised for 5 minutes and stopped because of pain in the chest. Figure 1A and B show superimposed averaged QRS waveforms at 1 and 5 minutes of exercise. Changes in the waveform indicate alterations in intraventricular conduction that are largely reversed post-exercise. Differences in the QRS waveforms were not noticed during the recording but are evident when the data sets are aligned accurately for comparison and calculation of the extent of change. Alignment is a crucial element of the method of analysis of vectors^2^.

Figure 2(A) charts the magnitudes of resultant vectors during 80 ms of depolarisation at 1 minute and 4.5–5 minutes of exercise. Progression of the magnitude of vectors from 6 to 21 ms of depolarisation is faster than e1, consequently the magnitude of resultant vectors is greater (RVM enhanced). However from 16 to 22.5 ms, progression is slowed and delayed by 1.5 ms relative to progression of the magnitude at e1 so that the magnitude of vectors from 24 to 27 ms is less than e1 (RVM reduced). Thereafter, the magnitude of vectors is increased, surpasses e1 at 30 ms and proceeds at the same rate as e1; consequently, the vector magnitude is greater than e1 for most of depolarisation until the apical vectors of the trend.

Figure 2(B) shows marked change between the differences in magnitude of consecutive resultant vectors at one minute and 4.5 to 5 minutes of exercise; from 19.5 to 25.5 ms of depolarisation, during slow progression of vector magnitude, and from 42 to 48 ms when slowing is barely perceptible. Figure 2(C,D) show substantial resolution of the changes while seated 6 minutes after exercise (pe6); reversible change is consistent with ischaemia.

MPS (tracer injected intravenously at 4 minutes of exercise) reported the following changes: (a) a large size, moderate-to-severe intensity, reversible anterior and apical defect; (b) a medium size moderate-to-severe intensity, predominantly-reversible inferior and inferior-lateral defect.

Slow progression of resultant vector magnitudes shown in Fig. 2(B) indicated: (a) at 19.5–25.5 ms, marked reduction in EMF located anterior-left-superior relative to the intersection of the orthogonal lead axes; (b) at 45–48 ms, moderate reduction in EMF located left-posterior-inferior.

These observations illustrate that slow progression of resultant vector magnitude relative to the first minute of exercise is the specific indicator of ischaemia; prominent scores for reduction or enhancement of RVM are consequential changes.

### Criteria for ischaemia derived from trends of resultant vector magnitude of volunteers

The trends of resultant vector magnitudes through depolarisation of healthy young volunteers, as those of patients, showed changes in the differences between magnitudes of consecutive vectors relative to the first minute at various stages of exercise. The differences between consecutive magnitudes were considered as proportions of the values at the corresponding times of the vectors at e1.

**Figure 3:**
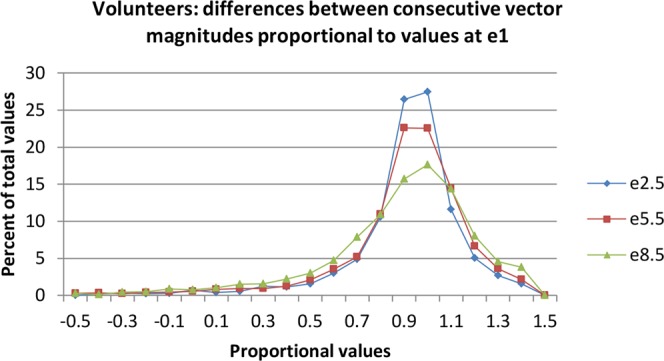
Frequency of differences between consecutive vector magnitudes of healthy young volunteers during exercise. Differences expressed as proportions of the values in the first minute of exercise. The percentile distribution of the values at 3 stages of exercise are shown: 2.5–3, 5.5–6, 8.5–9 minutes.

The range of changes in differences between consecutive vector magnitudes was defined in the group of volunteers, in order to identify changes that are not likely to be consistent with normality. The percentile frequency of approximately 2,300 proportional values of the differences (from −0.5 to 1.5) at each of 3 stages of the exercise test is shown in Fig. 3.

At 3 and 6 minutes of exercise proportional values below 0.7 occurred in 5% of the sample, and at 9 minutes below 0.65 in 6.3%. These values indicated significantly slower progression of resultant vector magnitude compared with e1 that are unlikely to be variants of normality. Therefore these values were used to identify ischaemia in the patients’ data.

Three or more contiguous vectors with significantly reduced proportions were considered because low values of one or two successive vectors occurred sporadically, caused by transient variation in alignment of data related usually to an inflexion in the trend of vector magnitudes.

### Application of the criteria for ischaemia

Using criteria for probability of myocardial ischaemia derived from the range of proportional reduction in the difference between consecutive vector magnitudes in contiguous vectors derived from the data of healthy young volunteers, a template of algorithms in Excel was developed to analyse the data of 114 patients for comparison with the results of MPS. Exclusion of 4 patients from 118 available for statistical analysis of vector magnitudes was due to uncertain identity of their MPS reports.

The analysis of each set of data was scrutinised to ensure validity of the results. The sensitivity and specificity of ischaemia identified by changes in electrical vectors by comparison with MPS at peak stress, were 88% and 71%, respectively; the latter due to more patients identified with ischaemia than by MPS. The results of all patients and those with and without past evidence of coronary artery disease (CAD) are given in Tables 1 and 2. For comparison, Tables 3 and 4 show correspondence of ST segment changes during ETT and MPS for reversible myocardial ischaemia.

**Table 1:** Indication of myocardial ischaemia during exercise by VCG and MPS.

Groups	MPS (+)	VCG (+)	MPS (−)	VCG (−)	MPS VCG (+)	MPS, VCG (−)	MPS (+) VCG (−)	MPS (−) VCG (+)
All Patients (n = 114)	25	49	89	65	22	63	3	26
Patients with past evidence of CAD^*^ (n = 42)	15	20	27	22	13	20	2	7
Patients without past evidence of CAD^*^ (n = 72)	10	29	62	43	9	42	1	20

MPS (+), reversible myocardial perfusion defect; MPS (−), no reversible perfusion defect; VCG (+), reversible changes in electrical vectors indicative of myocardial ischaemia; (VCG (−), no reversible changes in electrical vectors indicative of ischaemia.

*CAD: coronary artery disease.

**Table 2:** Identification of myocardial ischaemia during peak exercise by VCG with reference to MPS.

Groups	Sensitivity (95% CI)	Specificity (95% CI)
All Patients (n = 114)	88% (69–97)	71% (60–80)
Patients with past evidence of CAD^*^ (n = 42)	87% (60–98)	75% (55–89)
Patients without past evidence of CAD^*^ (n = 72)	90% (56–100)	69% (55–79)

Estimates of sensitivity and specificity were computed using MedCalc® statistical software.

CI: confidence interval.

^*^CAD: coronary artery disease.

Numbers rounded to the nearest integer.

**Table 3:** Identification of myocardial ischaemia during peak exercise by depression of the ST segment in 12-lead ECG during exercise with reference to MPS.

Groups	MPS (+)	ETT (+)	MPS (−)	ETT (−)	MPS ETT (+)	MPS, ETT (−)	MPS (+) ETT (−)	MPS (−) ETT (+)
All Patients (n = 114)	25	30	89	84	12	71	13	18
Patients with past evidence of CAD^*^ (n = 42)	15	15	27	27	9	21	6	6
Patients without past evidence of CAD^*^ (n = 72)	10	15	62	57	3	50	7	12

MPS (+), reversible myocardial perfusion defect; MPS (−), no reversible perfusion defect; ETT (+), reversible depression of the ST segment indicative of myocardial ischaemia; (VCG (−), no specific changes in the ST segment indicative of ischaemia.

*CAD: coronary artery disease.

**Table 4:** Correspondence of ST segment changes during ETT and MPS for reversible myocardial ischaemia.

Groups	Sensitivity (95% CI)	Specificity (95% CI)
All Patients (n = 114)	48% (28–69)	80% (70–88)
Patients with past evidence of CAD * (n = 42)	60% (32–84)	78% (58–91)
Patients without past evidence of CAD* (n = 72)	30% (7–65)	81% (69–90)

Estimates of sensitivity and specificity were computed using MedCalc® statistical software.

CI: confidence interval.

^*^CAD: coronary artery disease.

Numbers rounded to the nearest integer.

### Location of ischaemia

The location of ischaemia was derived from spatial orientation at e1 of vectors that were altered and reduction of EMF inferred from displacement during exercise. The cardiac structures affected by ischaemia were inferred from the spatial location of altered electrical forces. The location of ischaemia identified by VCG and MPS was similar in 15 of the 25 patients with reversible perfusion defects.

## Discussion

The results of extensive statistical investigation confirmed that the intensity and variation of changes in the magnitude and direction of electrical vectors during depolarisation is greater in the group of patients referred for assessment of inducible myocardial ischaemia by MPS than the group of healthy young volunteers (Table S1, Supplementary Material).

Multivariable analysis showed further that the magnitudes of planar vectors vary differently with stage of exercise in the early and late 40 ms periods of depolarisation. Durrer *et al*.^6^ found that in normal human hearts depolarisation progresses rapidly in a concentric manner from endocardial to epicardial myocardium in the first 40 ms, then slowly through epicardial myocardium to the pulmonary conus and posterior-basal aspects of the ventricles and interventricular septum. Therefore, the timing of changes in vectors broadly indicates regions of the ventricles that are affected. However, alteration of the magnitude and direction of vectors did not provide reliable criteria for identification of ischaemia induced by exercise.

Since the electrical field on the surface of the body is caused by electromotive forces generated by progression of ventricular depolarisation, sequential difference in the magnitude of resultant electrical vectors at a constant interval of 1.5 ms, is a measure of the rate of progression of depolarisation.

In an ischaemic region of myocardium, depolarisation spreads slowly resulting in irregular, asynchronous depolarisation^7^; consequently, its EMF and contribution to the resultant vector at that time are reduced. Therefore, we concluded that slow progression of the magnitude of resultant vectors is the prime indicator of hypoxia relative to cardiac work, not reduction of RVM as hypothesised initially.

**Figure 4:**
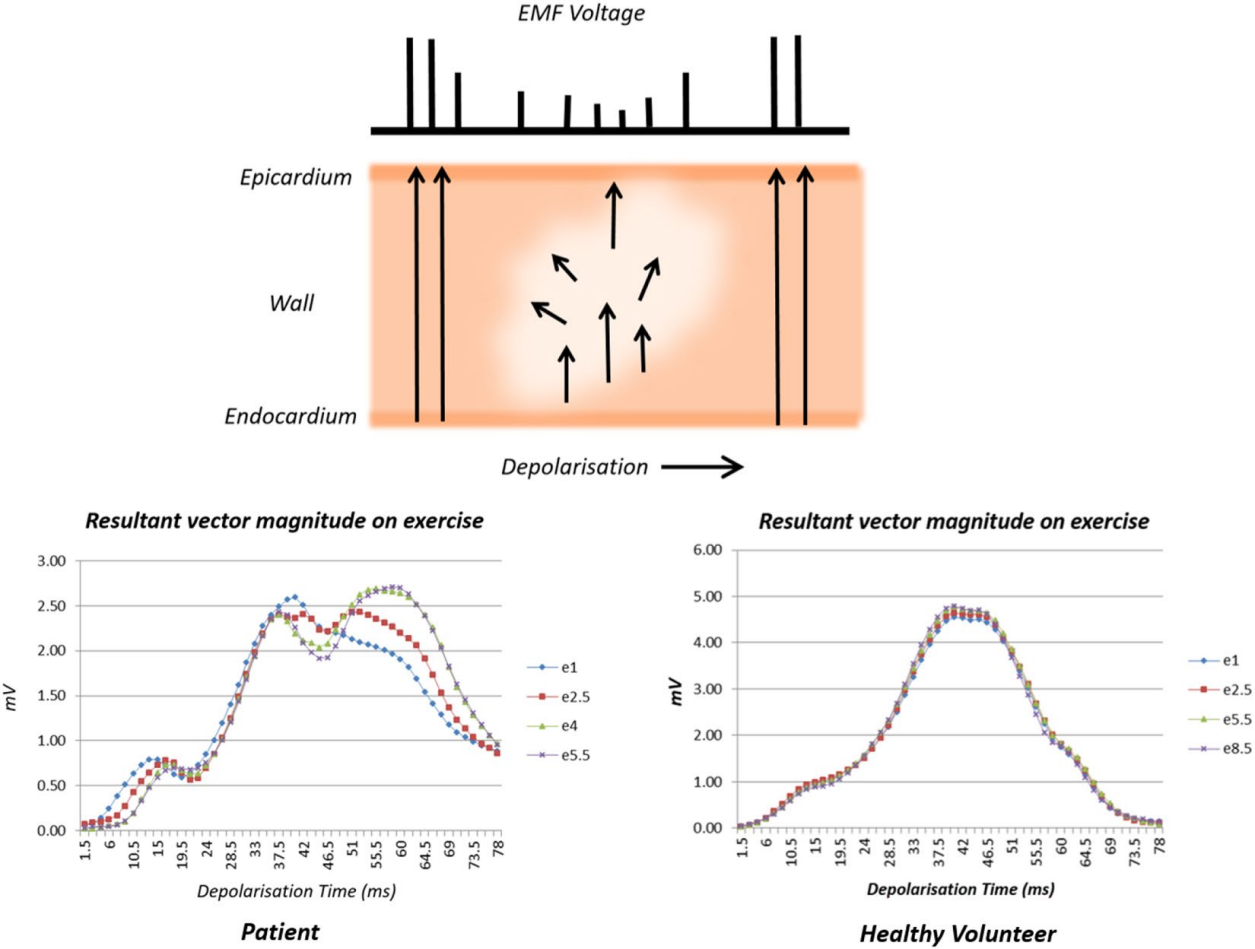
Electrophysiological Concept of the Study. The diagram illustrates a region of insufficient tissue perfusion for myocardial work in the process of depolarisation. Inadequate oxygenation results in reduction of EMF, which is reflected in the voltage of the electrical field at the surface of the body. The panel on the left shows trends of resultant vector magnitudes at stages of exercise in a patient with ischaemia. The trend at one minute of exercise (e1) is the reference for comparison of subsequent trends. At 4 minutes of exercise, slow progression of depolarisation at 4–9 ms delays the trend by 6 ms. Progression continues alongside e1 till 37.5 ms, then slows till 42 ms. Trends deviate with progressive delay as levels of stress increase indicating alterations in the sequence of depolarisation. By contrast, the panel on the right shows consistent trends of a young healthy volunteer from 1–9 minutes of exercise (e1 to e8.5).

Reduction in conductance of depolarisation leads to progression of depolarisation along pathways of greater conductance, thereby altering the sequence of depolarisation relative to the reference state and the magnitude of resultant vectors relative to cardiac work of the reference state, as demonstrated by the data displayed in Fig. 2. Even without affecting the sequence of depolarisation, irregular conductance of depolarisation will cause irregular distribution of EMF and displacement of the direction of resultant vectors away from the region of reduced conductance. Figure 4 is a diagrammatic representation of the electrophysiological changes that result in reduction of EMF, slow progression and block of depolarisation, and displacement of the direction of electrical forces.

Marked delay or block in the sub-endocardial Purkinje network will change the sequence of early depolarisation and distort the QRS waveform; such an event occurring very early is illustrated in Figs 1 and 2A. Trends of the magnitudes of resultant vectors changed in volunteers and patients as physical stress increased every 3 minutes. In patients, slow progression of depolarisation was observed for 1.5 to 4.5 ms, occasionally to 6 ms or longer. In volunteers, alteration in the trend was often evident in the first 10 ms of depolarisation, associated with marked change in direction. This implied variable facilitation of initial conduction pathways. The causes and significance of variation in the sequence of depolarisation with exercise in healthy subjects require further study.

Reduction in the difference between consecutive magnitudes of resultant vectors, expressed as a proportion of the relevant values at one minute of exercise below 95% of proportional values in healthy volunteers in at least three consecutive vectors, was the criterion for ischaemia applied on the records of patients. The results of exercise-induced ischaemia identified by changes in electrical vectors correlated well with reversible defects in perfusion shown by MPS (sensitivity 88%); however, more patients were detected by electrical vectors than MPS (Table 2), indicated by lower specificity (71%).

Considering the possibility that the lower specificity relative to sensitivity indicated a tendency to false positive detection of ischaemia by changes in electrical vectors, we noted that of 15 patients whose MPS showed only fixed defects of perfusion, electrical vectors identified ischaemia in 4. In 4 other patients MPS showed both fixed and reversible perfusion defects, and analysis of vectors identified reversible ischaemia in those 4 patients. These observations imply that analysis of vectors is probably more sensitive than MPS and specific for detection of myocardial ischaemia.

The results of changes in electrical vectors during exercise were clearly more comparable with MPS than depression of the ST junction and segment 80 ms later seen in the standard 12-lead ECG during ETT. ST changes indicative of myocardial ischaemia were observed in 25 of 114 patients tested with MPS but correspondence was found in 12 (Table 3); the sensitivity was 48% and specificity 80% (Table 4). Sensitivity was 60% for patients who had a past history of proven coronary artery disease but 30% for patients without a past history; specificity was 78% and 81%, respectively. The meta-analysis by Gianrossi *et al*.^8^ of 147 consecutively published reports comparing exercise induced ST depression with coronary angiography involving 24,074 patients showed wide variation in sensitivity and specificity, with mean values of 68%, SD 16% and 77%, SD 17%, respectively.

Reports on accuracy of MPS for coronary artery disease (stenosis greater than 50%) have been variable. In comparison with coronary angiography, a review of 15 studies^9^ incorporating 2,208 patients reported the sensitivity of SPECT to be 82%, specificity 76% and accuracy of 83% in detecting CAD. A meta-analysis reported sensitivity of 88% and specificity of 61% for SPECT^10^. A summary of 32 studies that included 4480 patients assessed for CAD showed mean sensitivity and specificity of 87% and 73%, respectively^11^. Evidently, MPS is not the ideal test of accuracy for detection of ischaemia by changes in electrical vectors induced by exercise; nevertheless it was the only reliable non-invasive test for stress-induced ischaemia done for all patients, with simultaneous recording of electrocardiographic signals for analysis of electrical vectors.

## Conclusion

The initial premise for detection of myocardial ischaemia, based on our previous report of a new method of analysing electrical cardiac vectors and its trial, focused on reduction of the magnitudes and changes in direction of resultant spatial vectors during exercise. The results of the study presented here confirmed this effect and showed that slow progression of depolarisation is the specific marker of intrinsic electrophysiological changes induced by ischaemia; other changes in vectors were secondary effects.

The question whether the specific indicator of ischaemia is more sensitive than MPS will be explored by prospective comparison of changes in electrical vectors during increased cardiac work with elective coronary angiography that is indicated clinically.

The method of analysing electrical vectors during exercise has the potential for developing a portable instrument for cost-effective, non-invasive investigation of patients with symptoms suggestive of myocardial ischaemia. When the accuracy of the method is known, the instrument may be considered for an epidemiological study to determine the onset of inducible ischaemia and its progression, in association with prevailing social and environmental conditions.

### Limitations

Selection subjects: the study was confined to male subjects because of culturally expected high frequency of refusal by women to accept the attachment of 20 electrodes on the torso required for two recording systems. The study relied on increasing workload by exercise so patients tested with coronary vasodilator or dobutamine were not analysed.

Technical matters: simultaneous recording of three ECG lead signals with minimal distortion by artefacts was a significant challenge. Accurate placement of electrodes was necessary for spatial location of ischaemia; inattention to the need for accuracy could have contributed to inconsistent location of ischaemia relative to MPS. Technical expertise of the person recording the orthogonal ECG signals is essential for enabling their computation, which can be done at a remote location.

## Supplementary information

Supplementary Information

## Data Availability

The datasets generated during and/or analysed during the current study are available from the corresponding author on reasonable request.
