# Supplementary material for: Changes in ventricular depolarisation vectors during exercise caused by regional myocardial ischaemia
Source: Sci Rep. 2019 Nov 8;9:16365. doi: 10.1038/s41598-019-52869-0 (PMC6841927; doi:10.1038/s41598-019-52869-0)
Supplement: Supplementary file 1 — Supplementary Information [file 41598_2019_52869_MOESM1_ESM.docx]

# SUPPLEMENTARY INFORMATION

Title: **Changes in ventricular depolarisation vectors during exercise caused by regional myocardial ischaemia**

Authors:

1. Cameruddin W. Vellani
2. Satwat Hashmi
3. Sadia Mahmud
4. Mohammad Yusuf
5. Safia Awan
6. Khawar Kazmi

# Supplementary Material-1 Univariate statistical analysis

**Table S1**: Comparison between patients (n=118) and healthy volunteers (n=51) relative to one minute of exercise: summed scores of changed magnitude and direction of contiguous vectors: Panels A, 2.5-3 min; B, 5.5-6 min; C, 8.5-9 min.

|  | **Patients**  **n; 118** | **Volunteers**  **n; 51** | ***p* value** | **Patients**  **n; 118** | **Volunteers**  **n; 51** | ***p* value** |
| --- | --- | --- | --- | --- | --- | --- |
| A | **e2.5: 1.5-19.5 m/s** | |  | **e2.5: 21-39** | |  |
| RVM -ve | -13.0 ± 13.4 | -6.2 ± 10.0 | 0.002 | -10.5 ± 14.9 | -4.9 ± 10.1 | 0.01 |
| RVM +ve | 8.4 ± 14.4 | 8.9 ± 13.7 | 0.83 | 6.3 ± 15.5 | 6.7 ± 13.4 | 0.85 |
| Direction | 7.3 ± 9.2 | 4.4 ± 5.7 | 0.03 | 3.7 ± 11.1 | 1.0 ± 1.9 | 0.09 |
|  | **e2.5: 40.5-58.8** | |  | **e2.5: 60-78** | |  |
| RVM -ve | -5.8 ± 12.8 | -1.2 ±2.5 | 0.01 | -4.9 ±11.9 | -2.5 ± 4.4 | 0.16 |
| RVM +ve | 16.6 ± 20.8 | 14.9 ± 15.8 | 0.60 | 20.1 ± 18.4 | 14.2 ±14.9 | 0.04 |
| Direction | 7.2 ± 17.1 | 2.4 ± 5.9 | 0.053 | 9.5 ± 16.6 | 4.1 ± 7.6 | 0.02 |

|  | **Patients**  **n; 117** | **Volunteers**  **n; 51** | ***p* value** | **Patients**  **n; 117** | **Volunteers**  **n; 51** | | ***p* value** |
| --- | --- | --- | --- | --- | --- | --- | --- |
| B | **e5.5: 1.5-19.5 m/s** | |  | **e5.5: 21-39** | | |  |
| RVM -ve | -24.5 ± 20.6 | -12.6 ± 13.4 | <0.001 | -22.8 ± 23.1 | | -8.2 ± 14.0 | <0.001 |
| RVM +ve | 9.8 ± 21.1 | 5.5 ± 9.0 | 0.16 | 11.7 ± 26.3 | | 6.6 ± 8.4 | 0.17 |
| Direction | 14.4 ± 17.3 | 7.5 ± 8.0 | 0.008 | 10.2 ± 20.2 | | 2.8 ± 7.5 | 0.01 |
|  | **e5.5: 40.5-58.8** | |  | **e5.5: 60-78** | | |  |
| RVM -ve | -12.7 ± 22.6 | -2.2 ± 6.5 | 0.001 | -4.4 ± 13.1 | | -2.6 ± 8.0 | 0.37 |
| RVM +ve | 42.1 ± 35.2 | 31.3 ± 21.2 | 0.04 | 43.9 ± 32.2 | | 32.3 ± 20.6 | 0.01 |
| Direction | 20.6 ± 25.4 | 4.3 ± 8.6 | <0.001 | 20.9 ± 20.4 | | 13.1 ± 13.3 | 0.01 |

|  | **Patients**  **n; 80** | **Volunteers**  **n; 51** | ***p* value** | **Patients**  **n; 80** | **Volunteers**  **n; 51** | | ***p* value** |
| --- | --- | --- | --- | --- | --- | --- | --- |
| C | **e8.5: 1.5-19.5 m/s** | |  | **e8.5: 21-39** | | |  |
| RVM -ve | -36.4 ± 22.7 | -27.4 ± 19.8 | 0.02 | -35.8 ± 28.5 | | -20.0 ± 23.9 | 0.001 |
| RVM +ve | 6.3 ± 10.4 | 3.0 ± 7.4 | 0.04 | 6.6 ± 13.2 | | 8.3 ± 10.3 | 0.44 |
| Direction | 20.7 ± 13.6 | 19.8 ± 16.5 | 0.74 | 12.9 ± 17.1 | | 11.3 ± 17.3 | 0.61 |
|  | **e8.5: 40.5-58.8** | |  | **e8.5: 60-78** | | |  |
| RVM -ve | -18.5 ± 31.2 | -5.1 ± 14.8 | 0.005 | -4.8 ± 19.6 | | -2.5 ± 9.4 | 0.42 |
| RVM +ve | 44.1 ± 30.9 | 44.7 ± 22.1 | 0.90 | 47.3 ± 28.2 | | 46.0 ± 20.8 | 0.77 |
| Direction | 29.0 ± 26.5 | 14.3 ± 19.2 | 0.001 | 31.1 ± 25.7 | | 19.7 ± 18.8 | 0.007 |

**Table S2:** Summed scores of changed magnitude and direction of contiguous vectors relative to one minute of exercise: of patients with ischaemia shown by MPS (MPS+ve) compared with scores of vectors of patients without past or current evidence of coronary artery disease (CAD - ve): Panels A, 2.5-3 min; B, 5.5-6 min; C, 8.5-9 min.

|  | **MPS+ve**  **n; 25** | **CAD –ve**  **n; 42** | ***p* value** | **MPS+ve**  **n; 25** | **CAD –ve**  **n; 42** | | ***p* value** |
| --- | --- | --- | --- | --- | --- | --- | --- |
| **A** | **e2.5: 1.5-19.5 ms** | |  | **e2.5: 21-39 ms** | | |  |
| RVM -ve | -13.5 ± 13.3 | -8.6 ± 9.1 | 0.08 | -10.4 ± 14.1 | | -5.4 ± 8.4 | 0.07 |
| RVM +ve | 12.3 ± 20.3 | 6.5 ± 8.3 | 0.10 | 10.9 ± 21.1 | | 4.3 ±8.4 | 0.07 |
| Direction | 11.7 ± 14.4 | 5.2 ± 6.5 | 0.01 | 9.6 ± 20.4 | | 2.1 ± 6.4 | 0.03 |
|  | **e2.5: 40.5-58.8** | |  | **e2.5: 60-78** | | |  |
| RVM -ve | -5.8 ±11.9 | -2.2 ± 4.0 | 0.08 | -5.1 ± 12.0 | | -3.4 ± 7.3 | 0.46 |
| RVM +ve | 16.6 ± 23.5 | 18.1 ± 19.6 | 0.78 | 21.3 ± 20.7 | | 21.4 ± 18.7 | 0.98 |
| Direction | 15.1 ±28.1 | 4.0 ±10.8 | 0.02 | 13.0 ± 25.3 | | 9.0 ± 12.5 | 0.38 |

|  | **MPS+ve**  **n; 25** | **CAD –ve**  **n; 42** | ***p* value** | **MPS+ve**  **n; 25** | **CAD –ve**  **n; 42** | ***p* value** |
| --- | --- | --- | --- | --- | --- | --- |
| **B** | **e5.5: 1.5-19.5 ms** | |  | **e5.5: 21-39 ms** | |  |
| RVM -ve | -22.3 ± 17.9 | -18.0 ± 14.1 | 0.27 | -20.8 ± 18.6 | -15.1 ± 15.5 | 0.08 |
| RVM +ve | 17.9 ± 30.4 | 5.1 ± 7.9 | 0.01 | 23.2 ± 38.0 | 7.1 ± 12.7 | 0.01 |
| Direction | 24.0 ± 31.0 | 9.1 ± 7.5 | 0.004 | 22.2 ± 36.1 | 4.0 ± 8.1 | 0.002 |
|  | **e5.5: 40.5-58.5** | |  | **e5.5: 60-78** | |  |
| RVM -ve | -10.6 ± 19.5 | -3.2 ± 7.0 | 0.03 | -4.9 ±14.6 | -2.7 ± 9.0 | 0.45 |
| RVM +ve | 46.4 ± 44.0 | 39.6 ± 29.9 | 0.45 | 53.7 ± 38.7 | 41.3 ± 28.4 | 0.13 |

| Direction | 31.5 ± 36.9 | 13.7 ± 19.2 | 0.01 | 24.2 ± 21.2 | 17.9 ± 19.4 | 0.21 |
| --- | --- | --- | --- | --- | --- | --- |

|  | **MPS+ve**  **n; 13** | **CAD –ve**  **n; 34** | ***p* value** | **MPS+ve**  **n; 13** | **CAD –ve**  **n; 34** | ***p* value** |
| --- | --- | --- | --- | --- | --- | --- |
| **C** | **e8.5: 1.5-19.5 ms** | |  | **e8.5: 21-39 ms** | |  |
| RVM -ve | -34.0 ± 23.1 | -33.3 ± 20.4 | 0.92 | -32.6 ± 27.9 | -31.1 ± 24.1 | 0.85 |
| RVM +ve | 7.1 ± 8.8 | 6.0 ± 8.5 | 0.68 | 8.3 ± 10.3 | 7.3 ± 16.4 | 0.83 |
| Direction | 19.6 ± 12.4 | 20.6 ± 12.0 | 0.81 | 14.7 ± 15.8 | 9.2 ± 13.5 | 0.24 |
|  | **e8.5: 40.5-58.8** | |  | **e8.5: 60-78** | |  |
| RVM -ve | -9.5 ± 21.4 | -12.4 ± 21.6 | 0.68 | -0.92 ± 2.2 | -2.3 ± 7.1 | 0.48 |
| RVM +ve | 35.0 ± 31.1 | 50.8 ± 33.4 | 0.14 | 45.3 ± 23.0 | 52.7 ± 31.2 | 0.44 |
| Direction | 32.6 ± 29.5 | 25.7 ± 23.8 | 0.40 | 39.6 ± 25.1 | 27.2 ± 24.3 | 0.12 |

# Supplementary Material-2 Multivariable statistical analysis

Multivariable Mixture Model analyses were conducted on SAS (SAS 9.3, 2002-2010 by SAS Institute Inc., Cary, NC, USA.) using PROC NLMIXED. A non-default option used was Double Dogleg optimization method to maximize the likelihood. This optimization technique works well for medium to moderately large optimization problems (Help and Documentation. SAS 9.3, 2002- 2010 by SAS Institute Inc., Cary, NC, USA). For each model, all possible *three* two-way interactions were assessed for inclusion in each part of the model. Level of significance was taken at 10%. P-values in the range (5% < p-value < 10%) are referred to as marginally significant, those (1% < p-value < 5%) as significant and < 1% as highly significant.

# Results of the second part of the mixture model

## Reduced RVM

A Probit log-skew-normal Mixture model was fitted for this outcome for each plane.

The second part of the Mixture Model considers (- Reduced RVM) > 0 as a continuous skewed distribution (log-skew-normal distribution). The results of the second part are reported as follows.

**Figure S1.** Histograms for Reduced RVM (labelled as RVM_negative) for all three planes respectively.

Horizontal plane


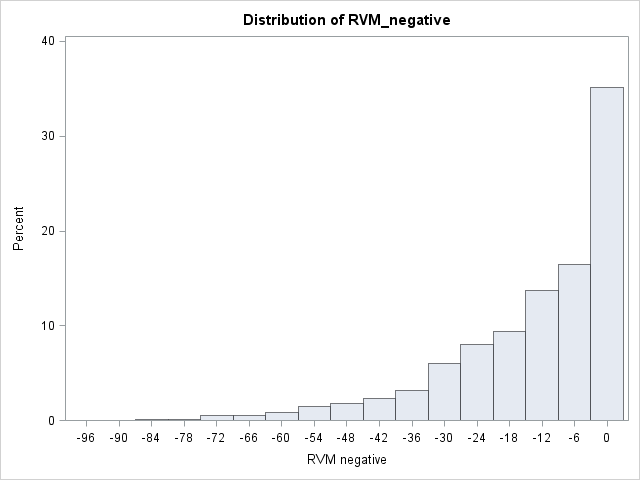


Frontal Plane


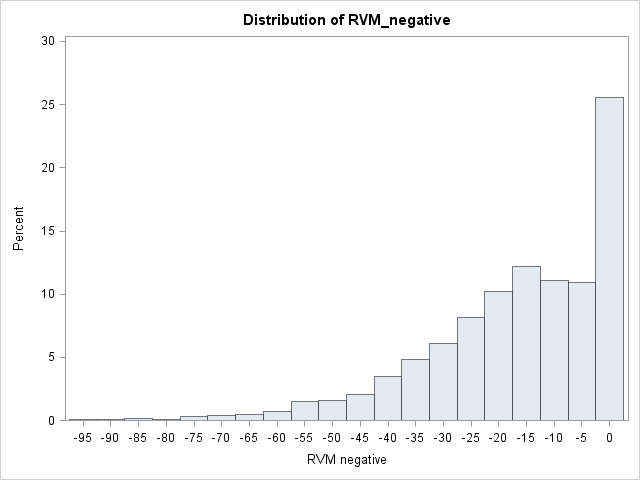


Sagittal Plane


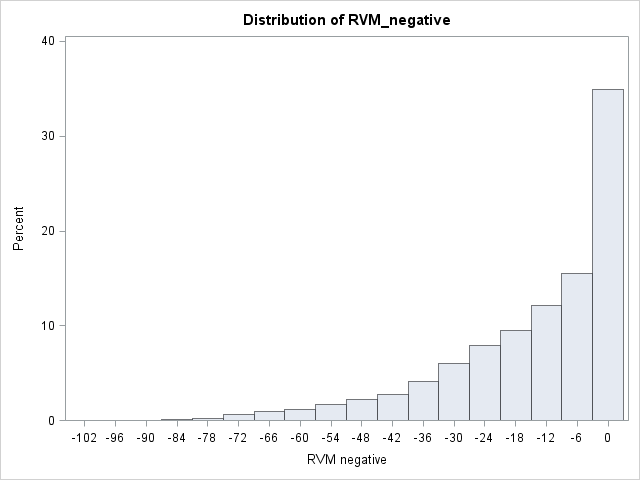


1. Horizontal plane

There is a highly significant interaction between exercise stage and depolarization cycle (Wald p-values < 0.0001), and that between group and depolarization cycle (Wald p-value= 0.0014) in this model component. The interaction between exercise stage and depolarization cycle is illustrated in Table S3.

**Table S3**. *Horizontal Plane***.** Difference in estimated mean of log *(-Reduced RVM> 0*) for 5.5 minutes and 8.5 min of exercise relative to that at 2.5 min of exercise respectively (*Diff*) (For either patients or volunteers).

| **Depolarization**  **cycle** | ***Diff* at 5.5 min**  **Wald p-value** | ***Diff* at 8.5 min**  **Wald p-value** |
| --- | --- | --- |
| 1st half | **0.3690**  **< 0.0001** | **0.7097**  **< 0.0001** |
| 2nd half | -0.0491  0.4922 | 0.0923  0.2288 |

*Bold Font indicate significant differences*

These results imply that for the first half of the depolarization cycle, for either patients or volunteers, there is a progressive increase in the *absolute* value of Reduced RVM< 0 from 2.5 min to 8.5 min of exercise. However, for the second half of the depolarization cycle, for either patients or volunteers, there seems to be no increase in the *absolute* value of Reduced RVM< 0 from 2.5 min to 8.5 min of exercise.

Analysis of the interaction between group and depolarization cycle indicates that the comparison between patients and volunteers is different for the two halves of depolarization cycle. For the first half of depolarization cycle, adjusting for the exercise stage, the mean log (- Reduced RVM) for patients is significantly greater than that for volunteers (Difference= 0.2835; Wald p-value = 0.0001). *This implies that for the first half of the depolarization cycle, at any stage of exercise,*

*the absolute value of Reduced RVM< 0 is greater for the patients compared to that of the*

*volunteers.* However, for the second half of the depolarization cycle, adjusting for the exercise

stage, the mean log (-Reduced RVM) for patients was not significantly different than that for the volunteers (Difference= -0.0018; p-value = 0.9830). *Hence for the second half of the*

*depolarization cycle, at any stage of exercise, there does not seem to be any difference between*

*the absolute value of Reduced RVM< 0 for the patients and the volunteers.*

1. Frontal plane

The parameter in the model corresponding to the group effect is highly significant. The mean log (- Reduced RVM) for patients was significantly greater than that for volunteers, adjusting for exercise stage and depolarization cycle (difference = 0.2718; Wald p-value < 0.0001). *This implies that at any stage of exercise, for either half of the depolarization cycle, the absolute value of Reduced RVM < 0 is greater for the patients compared to that of the volunteers.*

Moreover, there was a significant interaction between exercise stage and depolarization cycle. This interaction is illustrated in Table S4. These results indicate that for the first half of the depolarization cycle, for either patients or volunteers,

**Table S4**. *Frontal Plane*. Difference in estimated mean of log (*-RVM_negative > 0*) for 5.5 minutes and 8.5 min of exercise relative to that at 2.5 min of exercise respectively (For either patients or volunteers).

| **Depolarization**  **cycle** | ***Diff* at 5.5 minutes**  **p-value** | ***Diff* at 8.5 minutes**  **p-value** |
| --- | --- | --- |
| 1st half | **0.3453**  **< 0.0001** | **0.5873**  **< 0.0001** |
| 2nd half | **0.2177**  **0.0002** | **0.1864**  **0.0026** |

Bold Font indicates significant difference.

There seems to be a progressive increase in the *absolute* value of Reduced RVM < 0 from 2.5 min to 8.5 min of exercise. However, for the second half of the depolarization cycle, for either patients or volunteers, there is an increase in the *absolute* value of Reduced RVM < 0 from 2.5 min to 5.5 min with no further increase at 8.5 of exercise. In addition, we note that the increase in the *absolute* value of Reduced RVM < 0 from 2.5 minutes of exercise onwards is greater for the first half of the depolarization cycle as compared to the second half.

1. Sagittal plane

The mean log (-Reduced RVM > 0) for patients was significantly greater than that for volunteers, adjusting for exercise stage and depolarization cycle (difference = 0.3040; Wald p-value < 0.0001). This implies that at any stage of exercise, for either half of depolarization cycle, the *absolute* value of Reduced RVM < 0 is greater for the patients compared to that of the volunteers.

There is a significant interaction between exercise stage and depolarization cycle in this part of the model. This interaction is illustrated in Table S5. These results indicate that for the *first* half of the depolarization cycle, for either patients or volunteers, there seems to be a progressive increase in the *absolute* value of Reduced RVM < 0 from 2.5 min to 8.5 min of exercise. For the *second* half of the depolarization cycle, for either patients or volunteers, there is some evidence of an increase in the *absolute* value of Reduced RVM < 0 from 2.5 min to 5.5 min; however, the increase in the absolute value of Reduced RVM < 0 at 8.5 min of exercise relative to that at 2.5 min is more pronounced. In addition, we note that for the first half of the depolarization cycle the increase in the *absolute* value of Reduced RVM < 0 from 2.5 minutes of exercise onwards is greater as compared to that at the respective exercise stages for the second half.

**Table S5**. *Sagittal Plane***.** Difference in estimated mean of log *(-Reduced RVM > 0*) for 5.5 minutes and 8.5 min of exercise relative to that at 2.5 min of exercise respectively (*Diff*) (For either patients or volunteers).

| **Depolarization**  **cycle** | ***Diff* at 5.5 min**  **p-value** | ***Diff* at 8.5 min**  **p-value** |
| --- | --- | --- |
| 1st half | **0.3254**  **< 0.0001** | **0.6522**  **< 0.0001** |
| 2nd half | **0.1209**  **0.0658** | **0.1756**  **0.0168** |

Differences significant at 5% are highlighted in bold font Differences significant at 10% are shown in italics

## Enhanced RVM

Histograms for Enhanced RVM for all three planes are reported in Figure S2. A Probit log-skew- normal Mixture model was fitted for each plane.

**Figure S2.** Histograms for enhanced RVM (labelled as RVM_positive) for all three planes respectively.

Horizontal plane


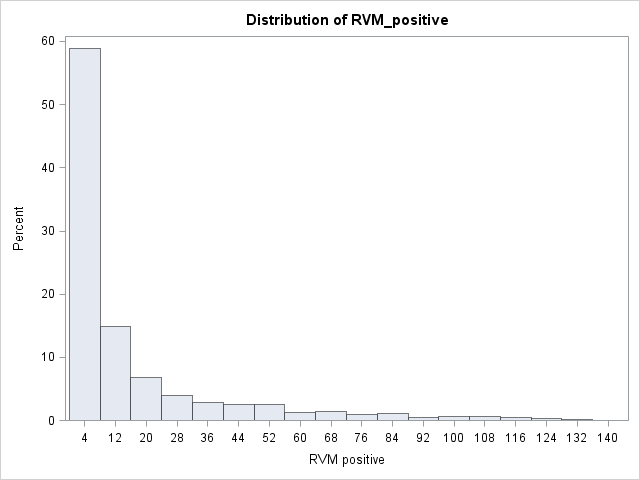


Frontal Plane


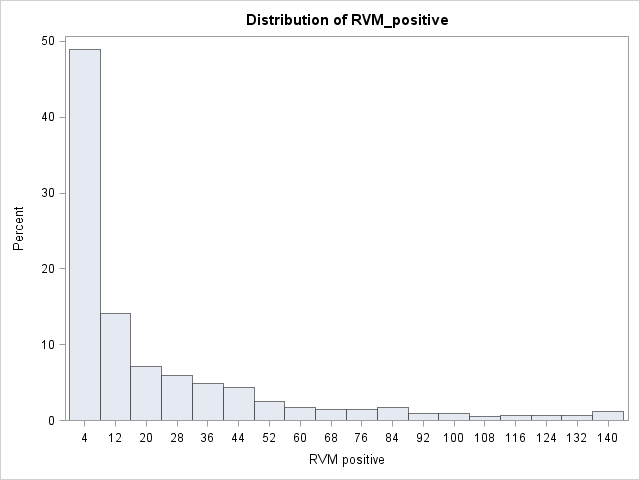


Sagittal Plane


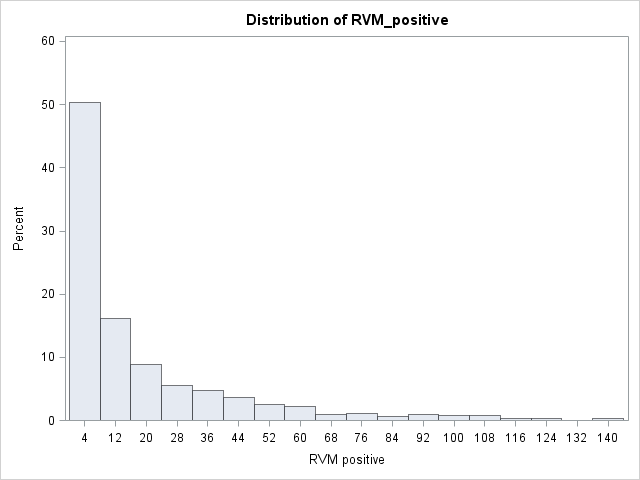


The second part of the Mixture Model considers (Enhanced RVM) > 0 as a continuous skewed distribution (log-skew-normal distribution). The results of the model fit for the second part are reported below.

1. Horizontal Plane

The effect of group was highly significant in the second part of the model; the mean log (Enhanced RVM) for patients was significantly greater than that for volunteers, adjusting for exercise stage and depolarization cycle (Difference = 0.2442; Wald p-value = 0.0026). *This implies that for either half of the depolarization cycle, and at any stage of exercise, Enhanced RVM >0 for patients was higher than that for the volunteers.*

In addition, there is a significant interaction between exercise stage and depolarization cycle in this part of the model (Wald p-values <0.01). This interaction is illustrated in Table S6.

**Table S6**. *Horizontal Plane.* Difference in estimated mean log (*Enhanced RVM > 0*) for 5.5 minutes and 8.5 min of exercise relative to that at 2.5 min of exercise respectively (For either patients or volunteers).

| **Depolarization**  **cycle** | ***Diff* at 5.5 min**  **Wald p-value** | ***Diff* at 8.5 min**  **Wald p-value** |
| --- | --- | --- |
| 1st half | 0.04287  0.7352 | -0.04207  0.7568 |
| 2nd half | **0.6458**  **< 0.01** | **0.9115**  **< 0.01** |

Differences significant at 5% are highlighted in bold font

For either patients or volunteers, there seems to be no change in Enhanced RVM > 0 with exercise for the first half of the depolarization cycle. However, for the second half of the

depolarization cycle, for either patients or volunteers there seems to be a progressive increase in Enhanced RVM > 0 with exercise.

1. Frontal plane

There is a highly significant interaction between group and depolarization cycle (Wald p-value

<0.01) in this part of the model (illustrated in Table S7). These results imply that for the first half of the depolarization cycle, at any stage of exercise, there does not seem to be any difference between Enhanced RVM > 0 for patients and volunteers. However, for the second half of the depolarization cycle, at any stage of exercise, Enhanced RVM > 0 is greater for patients compared to that of volunteers.

**Table S7**. *Frontal Plane*. Difference in estimated mean of log (*Enhanced RVM* > *0*) between patients and volunteers (*Diff*) at any stage of exercise.

|  | **Depolarization Cycle** | |
| --- | --- | --- |
|  | **1st half** | **2nd half** |
| *Diff* | -0.0831 | **0.2751** |
| p-value | 0.4714 | **0.0086** |

Differences significant at 5% are highlighted in bold font

In addition, there is a highly significant interaction between exercise stage and depolarization cycle (Wald p-values <0.01); this is illustrated in Table S8. These results imply that, for either patients or volunteers, for the first half of the depolarization cycle there seems to be no change in Enhanced RVM > 0 from 2.5 min to 5.5 min of exercise, whereas there is an increase in it at

8.5 min relative to that at 2.5 min of exercise. However, for the second half of depolarization cycle, for either patients or volunteers, there is a *progressive* increase in Enhanced RVM > 0

from 2.5 min to 8.5 min of exercise; this increase is greater than that for respective exercise stage for the first half of depolarization cycle.

**Table S8**. *Frontal Plane***.** Difference in estimated mean of log *(Enhanced RVM > 0*) at 5.5 minutes and 8.5 min of exercise relative to that at 2.5 min of exercise respectively (*Diff*), for either patients or volunteers.

| **Depolarization**  **cycle** | ***Diff* at 5.5 min**  **p-value** | ***Diff* at 8.5 min**  **p-value** |
| --- | --- | --- |
| 1st half | 0.0946  0.2575 | **0.3868**  **< 0.0001** |
| 2nd half | **0.6196**  **<0.0001** | **0.9240**  **<0.0001** |

Differences significant at 5% are highlighted in bold font

1. Sagittal plane

Interactions between exercise stage and group (Wald-p values equals to 0.0323 and 0.0533 respectively), and that between exercise stage and depolarization cycle (Wald p-values <0.0001) respectively are significant in this part of the model.

Interaction between exercise stage and group is illustrated in Table S9. At 2.5 minutes of exercise, for either half of depolarization cycle, there is some evidence that Enhanced RVM for patients is higher than that for volunteers. Moreover, for either half of depolarization cycle, at

5.5 and 8.5 minutes of exercise respectively Enhanced RVM is higher for patients than that for volunteers.

**Table S9.** *SAGITTAL PLANE***.** Difference in estimated mean of log (*Enhanced RVM* > *0*) between patients and volunteers for either half of the depolarization cycle (*Diff*).

| **Exercise Stage** | | | |
| --- | --- | --- | --- |
|  | **2.5 minutes** | **5.5 minutes** | **8.5 minutes** |
| *Diff* | 0.2164* | **0.5013** | **0.4827** |
| p-value | 0.0615 | **<0.01** | **<0.01** |

Differences significant at 5% are highlighted in bold font

*Difference is marginally significant; 5% < p-value < 10%).

Examining the effect of exercise stage on Enhanced RVM > 0, the two interactions, that between "exercise stage and group" and between "exercise stage and depolarization cycle" indicate that this effect depends on group and also on the two halves of depolarization cycle respectively.

Table S10 illustrates the effect of exercise stage on Enhanced RVM > 0.

**Table S10**. *SAGITTAL PLANE.* Difference in estimated mean of log(*Enhanced RVM* > *0*) between 5.5 and 8.5 min of exercise relative to 2.5 min of exercise respectively (*Diff*).

| **Group** | **Depolarization cycle** | **Exercise stage** | **Diff p-value** |
| --- | --- | --- | --- |
| Volunteers | 1st half  2nd half | 5.5 min  8.5 min  5.5 min  8.5 min | -0.0742 0.5795  0.0096 0.9445  **0.4465 0.0002**  **0.7221 <0.0001** |
| Patients | 1st half  2nd half | 5.5 min  8.5 min  5.5 min  8.5 min | **0.2107 0.0470**  **0.2759 0.0150**  **0.7314 <0.0001**  **0.9884 <0.0001** |

Differences significant at 5% are highlighted in bold font

For volunteers, for the first half of depolarization cycle, there is no change in Enhanced RVM at

5.5 and 8.5 min of exercise relative to that at 2.5 min. However, for volunteers, for the second half of depolarization cycle, there is a *progressive* increase in Enhanced RVM as they exercise from 2.5 min to 8.5 min. For patients, for the first half of depolarization cycle, there is an increase in Enhanced RVM at 5.5 min and 8.5 min of exercise relative to that at 2.5 min. Finally, for patients, for the second half of depolarization cycle, there seems to be a *progressive* increase in Enhanced RVM as they exercise from 2.5 min to 8.5 min. We note that the maximum increase in Enhanced RVM >0 with exercise occurs in patients for the second half of the depolarization cycle, particularly at 8.5 min of exercise relative to that at 2.5 min.

## Anticlockwise displacement of vector scores

Histograms for Anticlockwise displacement of vector scores for all three planes are reported in Figure S3. A Probit log-skew-normal Mixture model was fitted for each plane. The second part of the Mixture Model considers (-Anticlockwise scores) > 0 as a continuous skewed distribution (log-skew-normal distribution). The results of the model fit for the second part are reported below.

1. Horizontal Plane

For any stage of exercise, and either level of depolarization cycle, the estimated mean log (- Anticlockwise scores) for patients is significantly greater than that for volunteers (Difference = 0.6658; Wald p-value < 0.0001). This implies that the *absolute* value or the *magnitude* of anticlockwise scores < 0 is greater for patients compared to that for volunteers, adjusting for the stage of exercise and the level of depolarization cycle. Interaction between exercise stage and depolarization cycle (Wald p-values equals 0.0372 and 0.0002) is significant in this part of the model. This interaction is illustrated in Table S11.

**Figure S3**. Histograms for anticlockwise displacement of vector scores (labelled as Dir_ACL) for all three planes respectively.

Horizontal plane


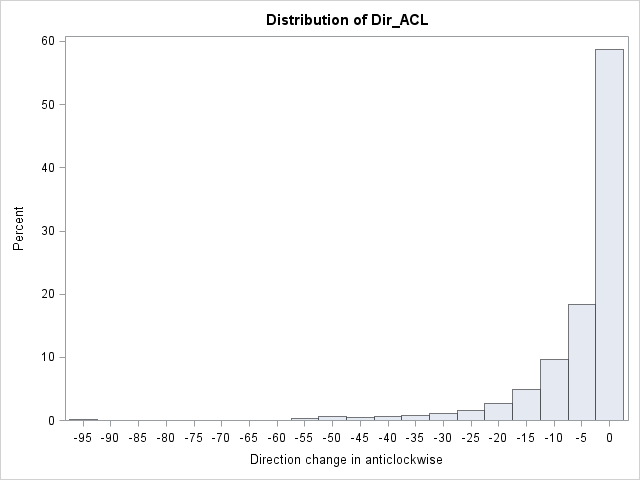


Frontal Plane


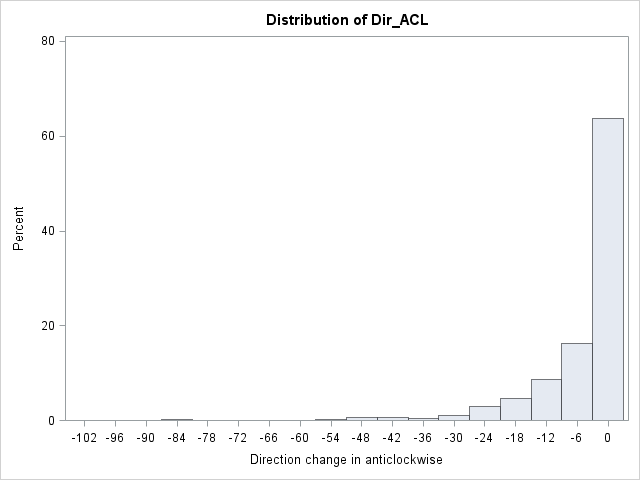


Sagittal Plane


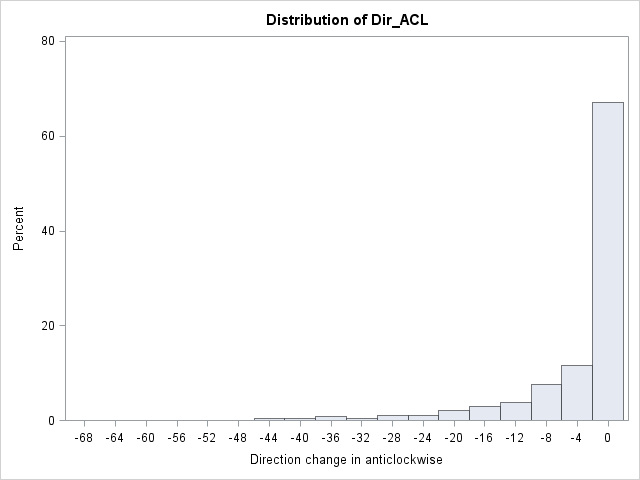


**Table S11**. *HORIZONTAL PLANE.* Difference in estimated mean of log *(-Anticlockwise scores >*

*0*) at 5.5 minutes and 8.5 min of exercise relative to that at 2.5 min of exercise respectively (*Diff*), for either patients or volunteers.

| **Depolarization cycle** | ***Diff* at 5.5 min p-value** | ***Diff* at 8.5 min p-value** |
| --- | --- | --- |
| 1st half | 0.1845* 0.0829 | **0.3810 0.0008** |
| 2nd half | **0.4739 <0.0001** | **0.9334 <0.0001** |

Differences significant at 5% are highlighted in bold font

*Difference is marginally significant; 5% < p-value < 10%).

Results in Table S11 indicate that for the *first* half of depolarization cycle, for either patients or volunteers, there is some evidence of an increase in the *magnitude* of Anticlockwise scores < 0 from 2.5 min to 5.5 min of exercise, and a considerable increase at 8.5 min relative to 2.5 min of exercise. However, for the *second* half of depolarization cycle, for either patients or volunteers, there is a *progressive* increase in the *magnitude* of Anticlockwise scores < 0 from 2.5 min to 8.5 min of exercise. *In conclusion, the interaction between exercise stage and depolarization cycle manifests itself as the increase in the magnitude of anticlockwise scores < 0 with increasing time of exercise being greater for the 2nd half than that for the first half of depolarization cycle.*

1. Frontal Plane

All possible three two-way interactions were significant in this part of the model; interaction between group and exercise stage (Wald p-values equal 0.0889 and 0.0013), that between group and depolarization cycle (Wald p-value=0.0216), and finally that between exercise stage and depolarization cycle (Wald p-values equal 0.0021 and 0.0006).

Interaction between 'group and exercise stage' and that between 'group and depolarization cycle' indicate that the comparison between patients and volunteers depends on both exercise stage and depolarization cycle. The results are reported in Table S12. For the first and second half of depolarization cycle at 2.5 minutes of exercise, and for the first half of depolarization cycle at 5.5 minutes of exercise, *magnitude* of Anticlockwise scores < 0 for patients was not different from that of volunteers.

**Table S12**. *FRONTAL PLANE*. Difference in estimated mean of log (*magnitude of Anticlockwise scores < 0*) between patients and volunteers (*Diff*).

| **Exercise Stage** | | | |
| --- | --- | --- | --- |
|  | **2.5 minutes 1st half**  **2nd half** | **5.5 minutes 1st half**  **2nd half** | **8.5 minutes 1st half**  **2nd half** |
| *Diff* | -0.1118 | 0.1449 | **0.3993** |
|  | 0.2123 | **0.4690** | **0.7234** |
| p-value | 0.4721 | 0.3292 | **0.0090** |
|  | 0.1882 | **0.0022** | **<0.0004** |

Differences significant at 5% are highlighted in bold font

Interaction between 'group and exercise stage' and that between 'exercise stage and depolarization cycle' indicate that the effect of exercise on *magnitude* of Anticlockwise scores <

0 depends not only on patients versus volunteers but also on the level of depolarization cycle.

**Table S13***. FRONTAL PLANE*. Difference in estimated mean of log (*magnitude of Anticlockwise scores < 0*) between 5.5 and 8.5 min of exercise relative to 2.5 min of exercise respectively (*Diff*).

| **Group** | **Depolarization cycle** | **Exercise stage** | **Diff p-value** |
| --- | --- | --- | --- |
| Volunteers | 1st half  2nd half | 5.5 min  8.5 min  5.5 min  8.5 min | -0.1392 0.3204  -0.1401 0.3376  0.2844* 0.0581  **0.3736 0.0160** |
| Patients | 1st half  2nd half | 5.5 min  8.5 min  5.5 min  8.5 min | 0.1175 0.2735  **0.3710 0.0012**  **0.5411 0.0000**  **0.8847 0.0000** |

Differences significant at 5% are highlighted in bold font

* Marginally significant difference (5% < p-value < 10%)

The results are reported in Table S13. For volunteers, during the first half of depolarization cycle, there is no change in *magnitude* of Anticlockwise scores < 0 at 5.5 min and 8.5 min relative to 2.5 min of exercise respectively. However, for volunteers, during the second half of depolarization cycle, there is some evidence of an increase in the *magnitude* of Anticlockwise scores < 0 at 5.5 min, and a considerable increase in *magnitude* of Anticlockwise scores < 0 at 8.5 min relative to 2.5 min of exercise. For patients, during the first half of depolarization cycle, there is no increase in *magnitude* of Anticlockwise scores < 0 at 5.5 min, but a considerable increase at 8.5 min relative to 2.5 min of exercise. For patients, during the second half of depolarization cycle, there is a substantial and seemingly *progressive* increase in

*magnitude* of Anticlockwise scores < 0 at 5.5 min and 8.5 min relative to 2.5 min of exercise

respectively. *We note that the largest increase in magnitude of Anticlockwise scores < 0 with*

*exercise occurs for patients during the second half of depolarization cycle.*

1. Sagittal plane

After adjusting for exercise stage and depolarization cycle, the mean log (-Anti- clockwise scores

> 0) for patients is marginally different from that of volunteers (difference = 0.1940; Wald p- value = 0.0680). This implies that at any stage of exercise, for either half of depolarization cycle, the *magnitude* of Anticlockwise scores < 0 has some tendency to be greater for patients than that for volunteers.

There is significant interaction between exercise stage and depolarization cycle in this part of the model (Wald p-values equal to 0.5019 and 0.0019 respectively). This interaction is illustrated in Table S14. These results imply that, for either patients or volunteers, for both the first and the second half of depolarization cycle, there is a *progressive* increase in the *magnitude* of Anticlockwise scores < 0 from 2.5 min to 8.5 min of exercise. However, we note that for the first half of depolarization cycle the increase in the *magnitude* of Anticlockwise scores < 0 from

2.5 minutes to both at 5.5 and 8.5 minutes of exercise is greater as compared to that at the respective exercise stages for the second half; with the increase at 8.5 min being about twice as large.

**Table S14**. *SAGITTAL PLANE*. Difference in estimated mean of log *(Magnitude of*

Anticlockwise scores < 0) at 5.5 minutes and 8.5 min relative to that at 2.5 min of exercise respectively (*Diff*), for either patients or volunteers.

| **Depolarization cycle** | ***Diff* at 5.5 min p-value** | ***Diff* at 8.5 min p-value** |
| --- | --- | --- |
| 1st half | **0.4964 < 0.0001** | **1.0775 < 0.0001** |
| 2nd half | **0.3805 0.0090** | **0.5128 0.0006** |

Differences significant at 5% are highlighted in bold font

## Clockwise displacement of vector scores

Histograms for clockwise displacement of vector scores for all three planes are reported in Figure S4. For Horizontal plane a Probit log-normal Mixture model is fitted (the skewness parameter is not significantly different from zero; Likelihood ratio test p-value=1.0). For Frontal and Sagittal planes, Probit-log-skew-Normal Mixture Models are fitted respectively. The results of the second part of the Mixture Model fit for each plane are reported below.

**Figure S4.** Histograms for clockwise displacement of vector scores (labelled as Dir_CL) for all three planes respectively.

Horizontal plane


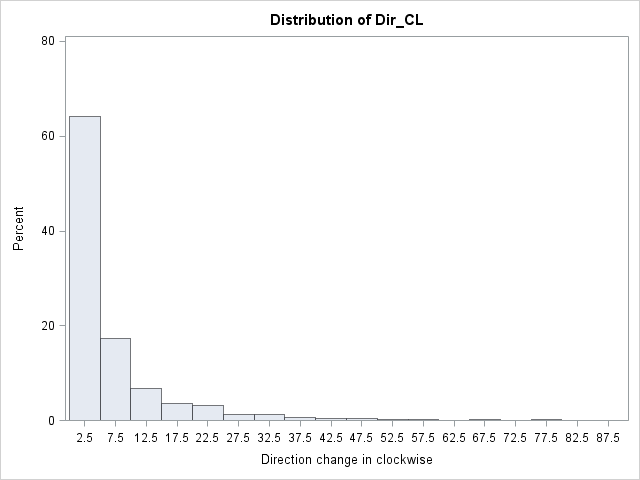


Frontal Plane


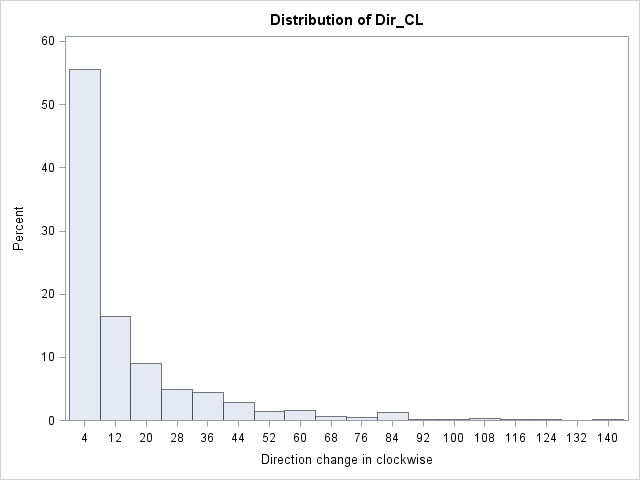


Sagittal Plane


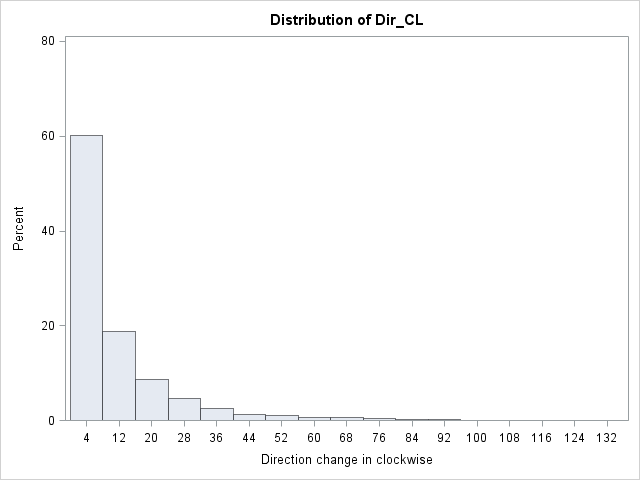


1. Horizontal plane

For the second part of the model there is *no* significant difference in the mean log (clockwise displacement of vector scores >0) between patients and volunteers adjusting for exercise stage and depolarization cycle (Wald p-value = 0.3515). Hence at any stage of exercise, for either half of depolarization cycle, there does not seem to be any difference between patients and volunteers with respect to clockwise scores > 0.

However, there is significant interaction between exercise stage and depolarization cycle (Wald p-values equal 0.3669 and 0.0008). This interaction is illustrated in Table S15. These results imply that for either patients or volunteers, for the first half of depolarization cycle, there appears to be a *progressive* increase in clockwise scores > 0 from 2.5 min to 8.5 min of exercise.

However, for the second half of depolarization cycle, for either patients or volunteers, there does not seem to be any change in clockwise scores >0 as subjects exercise from 2.5 to 8.5 min.

**Table S15**. *Horizontal Plane*. Difference in estimated mean of log(clockwise scores >0) at 5.5 minutes and 8.5 min of exercise relative to that at 2.5 min of exercise respectively (*Diff*), for either patients or volunteers.

| **Depolarization**  **cycle** | ***Diff* at 5.5 min**  **p-value** | ***Diff* at 8.5 min**  **p-value** |
| --- | --- | --- |
| 1st half | **0.2690**  **0.0021** | **0.6830**  **< 0.0001** |
| 2nd half | 0.1395  0.2225 | 0.1466  0.2501 |

Differences significant at 5% are highlighted in bold font

1. Frontal plane

At any stage of exercise, for either half of depolarization cycle, the mean log (clockwise scores

>0) for patients is marginally different from that of volunteers (difference =0.1447, Wald p- value=0.0674); the positive sign of the difference indicates tendency of clockwise scores > 0 for patients to be greater than that for volunteers.

For either volunteers or patients, at any stage of exercise, mean log (clockwise scores > 0) is significantly greater for the 2nd half of depolarization cycle than that for the first half (difference

= 0.2392; Wald p-value < 0.0001).

For both volunteers and patients, for either half of depolarization cycle, the mean log (clockwise scores > 0) at 5.5 min (difference= 0.4844; Wald p-value <0.0001) and 8.5 min (difference= 0.8868; Wald p-value <0.0001) are significantly higher than the mean log (clockwise scores > 0) at 2.5 min of exercise. These results indicate a *progressive* increase in clockwise scores > 0 as subjects exercise from 2.5 to 8.5 min.

iii) Sagittal plane

There is a marginally significant interaction between group and depolarization cycle (Wald-p value = 0.0964) in this part of the model. The marginal interaction between group and depolarization cycle indicates that the comparison between patients and volunteers differs somewhat for the two levels of depolarization cycle. This interaction is illustrated in Table S16. These results imply that for any stage of exercise and either half of depolarization cycle, (clockwise scores > 0) for patients are higher than that for volunteers, with the difference being *more* pronounced for the first half of depolarization cycle.

**Table S16**. *Sagittal Plane*. Difference in estimated mean of log (*clockwise scores* > *0*) between patients and volunteers (*Diff*), for any stage of exercise.

|  | **Depolarization Cycle** | |
| --- | --- | --- |
|  | **1st half** | **2nd half** |
| *Diff* | **0.5297** | **0.3554** |
| p-value | **<0.0001** | **0.0002** |

Differences significant at 5% are highlighted in bold font

In addition, there is a highly significant effect of exercise stage in this part of the model (Wald p- values <0.0001). The difference in estimated mean log (clockwise scores >0) at 5.5 min of exercise relative to 2.5 min is 0.3092 (Wald p-value < 0.0001), whereas that between 8.5 min of exercise relative to 2.5 min is 0.6047 (Wald p-value < 0.0001). This implies a *progressive* increase in (clockwise scores > 0) from 2.5 min to 8.5 min of exercise, for both patients and volunteers, at either half of depolarization cycle.
